# Hashimoto Encephalopathy: A Rare Intricate Syndrome

**DOI:** 10.5812/ijem.4174

**Published:** 2012-04-20

**Authors:** Juraj Payer, Tomas Petrovic, Lubomir Lisy, Pavel Langer

**Affiliations:** 1 Clinic of Internal Medicine, Faculty of Medicine, Comenius University, Faculty Hospital Ruzinov, Bratislava, Slovakia; 2 Clinic of Neurology, Slovak Medical University, Bratislava, Slovakia; 3 Institute of Experimental Endocrinology, Slovak Acadeamy of Sciences, Bratislava, Slovakia

**Keywords:** Hashimoto’s Encephalitis, Vasculitis, Glucocorticoids

## Abstract

Recently, several patients have been reported with various signs of encephalopathy and high thyroid antibody levels together with good responsiveness to glucocorticoid therapy. Despite the various clinical presentations, these cases have been termed “Hashimoto encephalopathy” (HE). Although all of the pathogenic components have not yet been clearly elucidated, it is believed that brain vasculitis and autoimmunity directed against common brain-thyroid antigens represent the most likely etiologic pathway. The most common clinical signs include unexplained or epilepsy-like seizures resistant to anti-convulsive treatment, confusion, headaches, hallucinations, stroke-like episodes, coma, impairment of cognitive function, behavioral and mood disturbance, focal neurological deficits, disturbance of consciousness, ataxia, and presenile dementia, together with the presence of high thyroid antibody levels, especially against thyroperoxidase (*TPOab*). In most cases, the thyroid function is normal or decreased; the thyroid function is rarely increased. The examination of the cerebrospinal fluid, EEG, MRI, SPECT, and neuropsychological examinations are primarily used as diagnostic tools. Most cases showed neural symptoms for months before the acute onset; in some cases, a dramatic acute onset was described. Once the diagnosis is made, corticosteroid treatment usually provides a dramatic recovery. The authors also present a short review of literary cases reported in last decade.

## 1. Introduction

The term “Hashimoto encephalopathy” (HE) was used perhaps for the first time in 1991 by Shaw (1), who collected five cases with similar symptoms such as seizures, disorientation, frequent episodes of alternating hemiparesis, high protein levels in the cerebrospinal fluid and electrocardiographic abnormalities. However, these patients also had hypothyroidism and positive thyroid antibodies. However, in 1966, a case was described of a 63-year-old man who had seizures, disorientation, frequent episodes of alternating hemiparesis, high protein levels in the cerebrospinal fluid, electrocardiographic abnormalities and biopsy-confirmed Hashimoto thyroiditis (2) Because several cases were later published that showed a similar outcome but with entirely different clinical presentations, the question has been raised whether HE is a true syndrome. The same authors concluded that several patients presented with various signs of encephalopathy and high thyroid antibody levels together with the responsiveness to glucocorticoid therapy and that such convergence seems unlikely to result from chance (3). There also appears to be no evidence of any specific pathogenic role for thyroid antibodies in the origin of such encephalopathy, and several authors hypothesized that these antibodies are only markers of a possibly unrelated autoimmune disease affecting the brain. The term HE is now commonly used for the few hundred patients published so far, whereas some other terms such as “myxedema madness” (4), “encephalopathy associated to autoimmune thyroid disease” (5) or “steroid-responsive encephalopathy with antibodies to thyroperoxidase” (SREAT) (6) have been mostly abandoned.

## 2. Pathogenesis

Thus far, it appears that there are several pathogenic components of this life-threatening disease and, although several of them still should be elucidated, the decisive role of the autoimmune component in the pathogenesis of this disease appears to generally accepted, as supported by some common circumstances such as the majority of female patients, the fluctuating course of the disease and the association with other autoimmune diseases. In general, when considering the great variety of symptoms, the mechanisms that are thought to underlie HE include disseminated encephalomyelitis and/or also autoimmune general cerebral vasculitis (1, 7, 8); these mechanisms possibly result in a broad spectrum of functional symptoms and multifocal abnormalities resulting from impaired cerebral perfusion and metabolism (9).

In several patients, an increased level of thyroid antibodies, i.e., those against thyroperoxidase (TPoab) but sometimes also those against thyroglobulin (TGab) or the thyrotropin receptor (TRab), has been found and in some patients, even such an occasional finding became a decisive input that led to a final diagnosis. In the great majority of patients, the thyroid function is normal or decreased, but in some rare cases listed below, the thyroid function was found to increase. Although some authors did not find any evidence of a causative link between thyroid autoimmunity and encephalitis (10), some others also found thyroid antibodies in the cerebrospinal fluid of HE patients. Therefore, speculations about a possible pathogenic role of the antibodies was introduced, as well as the possibility of intrathecal origin was suggested (11). The level of the antibodies considerably decreased in parallel with the clinical improvement of the patient (12).

Few years ago, a new autoimmune antigen, amino terminal of alpha-enolase (NAE), was found in the brain of HE patients, and a high level of antibodies against this antigen was also found in HE patients (13), whereas such antibodies were not detected in patients with other neurological diseases. Moreover, in the serum of two HE patients, anti-neuronal antibodies were found that reacted with the 36-kDa antigenic protein present in a soluble fraction obtained from the human cerebral cortex, and such autoantibodies were suggested to be associated with the pathogenesis of HE (14). The presence of NAE has been also found in 83.3% (15) and 68% of HE patients (16), thus confirming its high specificity for HE and emphasizing that, together with thyroid antibodies, NAE is a useful diagnostic marker for HE.

It is also believed that several species of anti-neuronal antibodies recently detected in HE patients play a role in the pathogenesis. Cerebrospinal fluid IgG from HE patients bound to enzymes in the human CNS such as dimethylargininase-I and two isoforms of aldehyde reductase-I, which are distributed in endothelial cells and neurons in the normal human CNS. The autoimmune response to these enzymes may lead to vascular and/or neuronal damage, which are two major mechanisms involved in the pathogenesis of HE (17). However, possibly because of the extremely varied clinical presentation, it has been repeatedly claimed that the pathogenesis of HE remains unknown, although brain vasculitis and autoimmunity directed against common brain-thyroid antigens represent the most likely etiologic pathway. Nevertheless, the interrelations between the clinical presentation, thyroid disease, auto-antibody pattern and brain pathology should be further clarified (18).

Some pathogenetic relationship between Hashimoto thyroiditis and HE has been also derived from the finding of impaired cerebral perfusion by SPECT (single photon emission computed tomography) in a group of 41 patients with Hashimoto’s thyroiditis but with a normal detailed neurological history and normal actual neurological findings as compared with 35 healthy controls (19). Hence, it was suggested that the impaired cerebral perfusion observed in patients with HE could result from cerebral vasculitis possibly present in several patients with autoimmune (Hashimoto’s) thyroiditis and thus could be considered as a pathogenetic model of HE. The decisive role of SPECT examination for HE diagnosis has been recently supported by the observation of clinical features in five patients, including the interrelation with clinical and immunological parameters in one patient who had the longest-standing HE that the authors had seen (20).

## 3. Clinical presentation

HE is a relatively rare condition with a broad range of clinical presentation. Thus, there is a high risk that patients with this serious disease will be misdiagnosed and thus even mistreated, sometimes for a considerably long time. Because the finding of TPOab in blood is one of the most frequent signs accompanying HE, it is generally recommended as a powerful diagnostic tool in all cases of unexplained fluctuating encephalopathy. The most frequently observed signs include epilepsy-like seizures resistant to anticonvulsive treatment, confusion, headaches, hallucinations, stroke-like episodes, coma, impairment of cognitive function, behavioral and mood disturbance, focal neurological deficits, disturbance of consciousness, ataxia, and presenile dementia.

Although the majority of described cases showed neural symptoms for months before the acute onset, in some cases a dramatic acute onset appeared. A review of 30 patients revealed two types of clinical presentation, e.g., that with acute or insidious onset (21). In general, this view seems to be supported by an overview of several patients as summarized below. However, a third type of onset and clinical course of a variety of neurologic complications was recently defined as “relapsing-remitting manner including cognitive deterioration and psychiatric illness” (8).

HE appears in all age groups including children (22), and as much as approximately 70 to 80 percent of the patients are women and girls. It was repeatedly underlined that, especially in children, several cases of such encephalopathy remained unrecognized for a considerable time (8, 23, 24). However, in several cases, a clear thyroid disorder, mostly presenting as hypofunction, could provide useful streamlining diagnostic guidance in patients with various neuropsychiatric signs. Such signs usually continue after the thyroid treatment or even show a worsening that is generally considered to be one of the most important signs of HE, as the specific neuropsychiatric problems usually accompanying each thyroid disorder disappear after treatment with thyroxine.

## 4. Diagnosis

Some useful reports have appeared regarding the frequency of individual symptoms and/or laboratory findings in patients with HE. Chong et al. (3) summarized 105 cases and concluded that in most cases, the diagnosis was based on the disturbed consciousness, negative finding of bacterial or viral infection in the cerebrospinal fluid, and high level of thyroid antibodies, with latter found in 100% of the cases, although the antibody level usually did not appear to be correlated with the severity of the illness. Moreover, a high protein level in the cerebrospinal fluid appeared in 78% of cases, abnormal electroencephalography in 98% and various mostly nonspecific abnormalities on magnetic resonance imaging in approximately 50%. Magnetic resonance revealed ischemic areas, multiple tumors, granulomas or various degenerative processes in 60% of the cases, and SPECT examinations showed decreased perfusion in the cortical areas or basal ganglia (25). From 1995 to 2003, 20 patients with Hashimoto encephalopathy were examined (26) and tremor was found in 16, transient aphasia in 16, myoclonus in 13, walking impairments in 13, seizures in 12 and sleeping disorders in 11 of them.

Cerebrospinal fluid analysis, electroencephalography, and neuroimaging studies do not show consistent findings to support the diagnosis. Physicians’ awareness of this complication is of great importance because most patients respond dramatically to corticosteroid therapy. Moreover, early recognition might also prevent a costly diagnostic work-up in patients with unexplained encephalopathy. In one patient, a follow-up for the IgG level in the CSF was found to be useful; after the IgG was found to be increased, the treatment was repeated with a partial clinical improvement and decline in the CSF level of IgG. Following high-dose steroid treatment, the patients’ clinical condition stabilized and a CSF analysis showed even further IgG decline (27). Thus far, approximately 150 cases have been published, although this disease probably remains under-diagnosed because it is not yet generally known and there were also presumably several additional cases that simply were not published at all. As clearly follows from the literature, an unusually wide variety of symptoms at presentation effectively obscures the basic pathogenetic process of HE. Because thyroid antibodies recently became considered as a useful marker for HE, it is recommended to check for their elevation not only in the blood but also in the CSF, particularly in cases presenting the triad of encephalopathy with EEG slowing and increased protein level in the CSF as well as in all unexplained cases with a variety of nonspecific neuropsychiatric symptoms, generalized or partial seizures, hallucinations, or status epilepticus.

The MRI manifestations of HE can vary from normal appearance, ischemic lesions, demyelination, and vasogenic edema to atrophy. The diverse MRI features of HE reported in the literature make it difficult to understand the pathological process and monitor the prognosis. To investigate the dynamic changes of MRI manifestations in HE, two cases of HE were retrospectively analyzed with a series of longitudinal MRI data. Although similar acute ischemic manifestations were observed at the onset of HE in both cases, at follow-up, we observed different evolutions of HE on MRI between the two cases, which might partially account for the diversity of MRI findings (for example, a certain stage of HE). The clinical and MRI findings at follow-up also indicated that early treatment contributed to the recovery of the lesions (28).

## 5. Treatment

Once a firm HE diagnosis is made, corticosteroid treatment usually provides a dramatic recovery, but several adverse outcomes, relapses and temporary or permanent spontaneous remissions have also been reported. At the same time, the high effectiveness of corticoid treatment in nearly all HE patients strongly supports an common autoimmune origin. Even in the absence of diagnostic serological findings, clinical improvement with corticosteroids may be provide the only evidence of autoimmune encephalopathy (29). However, it is always necessary to consider the possible adverse effects of corticosteroid therapy (30). Additionally, a case of HE was described that improved only after intravenous immunoglobulin treatment (31).

## 6. Selected Cases of HE Published Within the Last Decade (2001–2011)

Useful information about the symptomatology and diagnostic problems of HE could be obtained from the overview of several selected cases published within the last decade (Table 1).

**Table 1:** Overview of Selected Cases of Hashimoto’s Encephalopathy

Author	Case	Clinical Presentation	Finding
Mahmud, *et al*. (2003) (10)	14-year-old girl	visual and auditory hallucinations	*MRI* a- disseminated foci in frontal lobe
			*EEG* a- area of decreased perfusion
Yamanouchi, *et al*. (2006) (32)	9 cases of infants	status epilepticus, hyperpyrexia, prolonged impairment of consciousness, stereotypic movements, instability of mood, catalepsy.	*MRI* a- transient edema in both frontal lobes
			*EEG* a - attenuated cerebral perfusion in the frontal lobes (Serial studies - atrophic changes in the frontal lobes)
Muhle, *et al*. (2009) (34)	15-year-old girl	cerebral seizures	*MRI* a - cortical edema
Azdin-Ozemir, *et al*. (2006) (36)	37-year-old male	severe multifocal status epilepticus, ataxia, semirhythmic convulsions	*MRI* a - impaired signals in precentral cortex
George, *et al*. (2007) (39)	35-year-old woman	progressive spastic paraparesis	*MRI* a- hyperintense signal in the spinal cord and brain
			*muscle biopsy* - perivascular lymphocytes around endomysial vessels
Shindo,*et al*. (2007) (43)	37-year-old woman	persistent high fever, confusion, neck stiffness, anterograde and retrograde amnesia and disorientation	*MRI* a- nearly symmetric high signal intensity areas in the bilateral mesial temporal lobes
			*positive antibodies* - anti-NAE a in the serum and against glutamate receptor (GluR) epsilon2 in the serum and CSF a
Nakagawa, *et al*. (2007) (49)	41-year-old woman	progressive severe gait ataxia, slurred speech	*MRI* - normal
			*EEG* a- diffuse slow-wave activities (7–8 Hz) without any epileptic discharge
			*positive antibodies* - anti-NAE
Aquino, *et al*. (2009) (51)	42-year-old woman	fever, insomnia, cramps, tremors, athralgia, back pain and paraesthesia of hands, behavioral changes, loss of the nails of her right hand, lesions on back and face compatible with excoriation	*MRI* - normal
			*EEG* a - normal
			*EEG* - normal
			*CSF* a - normal
Chang, *et al*. (2010) (54)	48-year-old woman	progressively declining cognitive function without neurological focal deficit	*MRI* - cortical and subcortical white matter demyelination
Chong, *et al.* (2011) (58)	53-year-old woman	generalized seizures, rapid decline in cognitive function, increasing dependency and gradual change in personality	*MRI* - deep white matter ischemic changes, mild cortical atrophy
Saito, *et al*. (2002) (61)	23-year-old woman	generalized convulsions, disturbance of consciousness, cognitive deficit of short-term memory	*MRI* - normal
			*CSF* - elevated protein level
			*EEG* - global decrease of cerebral perfusion
			*EEG * - diffuse slowing of the background rhythm without any signs of epileptic activity (history of Graves’ disease with normal thyroid function at hospital admission)
Sakurai, *et al*. (2008) (63)	79-year-old woman	unconsciousness and convulsion following mental deterioration	*positive antibodies* - TRab a (Basedow’s disease diagnosis), TPOab a, TGab a and anti - NAE a
			*EEG* a - abnormal findings potentially indicating periodic synchronous discharge
			*MRI* a - cerebral atrophy
			*EEG* a - overall decrease in the accumulation of 99mTc in the cerebrum
Payer, *et al*. (2009) (67)	27-year-old woman	4-month history of thyreotoxicosis, febrile status, convulsions, psychotic symptoms, altered consciousness with the need of artificial lung ventilation, deterioration of neuropsychological status after repeated attempts to withdraw corticosteroids	*MRI* a - normal
			*EEG* a - normal accumulation in both hemispheres
			*CSF* a - increased level of proteins
			*EEG* a - episodes of rhythmic delta activity
			*positive antibodies* - TPOab a, TGAb a, TRAb a
			*Laboratory tests* - low TSH a and high fT4 and fT3 levels
			*thin needle thyroid biopsy* - signs of chronic exacerbated Hashimoto thyroiditis

^a^Abbreviations: MRI, Magnetic resonance imaging; SPECT, Single-photon emission computed tomography; EEG, Electroencephalography; CSF, Cerebrospinal fluid; TSH, Thyroidstimulating hormone; TRab, Anti TSH receptor antibodies; TPOab, Anti-thyroid peroxidase antibody; TGAb, Antithyroglobulin antibody; Anti-NAE, Autoantibodies against the NH2-terminal of a-enolase

Some peculiar cases were observed in children, and some authors supposed that, in children, this encephalopathy is likely under-diagnosed (8). One unusual patient was a 14-year-old girl suffering from visual and auditory hallucinations since the age of nine years, which resulted in fear and a bad mood. She had negative EEG findings and was treated by psychotropic drugs for 6 months. MR showed disseminated foci in the frontal lobe, and SPECT showed decreased perfusion in the left temporal lobe, basal ganglia and frontal lobes. Because her twin sister had autoimmune (Hashimoto) thyroiditis, the thyroid of this patient was finally checked, and severe hypothyroidism and positive TPOab were observed. After one month of treatment with thyroxine, partial improvement appeared, but definite improvement was found after long-term treatment with thyroxine and prednisone (10). Japanese authors reported nine cases of infants with acute encephalopathy involving the bilateral frontal lobes, with convulsive status epilepticus and hyperpyrexia followed by a prolonged impairment of consciousness for 2–20 days. Some of the infants also exhibited stereotypic movements, instability of mood, or catalepsy. Transient postictal edema in both frontal lobes was suggested by diffusion-weighted MRI, and SPECT showed attenuated cerebral perfusion in the frontal lobes on the tenth day after onset or subsequently. Serial studies disclosed atrophic changes in the frontal lobes. All patients showed regression or retardation of the motor and verbal functions. The recovery of intellectual deficit was slower and less prominent than that of motor dysfunction. These unique features suggest that the frontal lobes are the focus of this novel HE subtype, which is tentatively called “acute infantile encephalopathy”. After recovery from consciousness, all of the patients manifested regression of verbal function and lack of spontaneity (32). Additionally, a 6-year-old girl presented with progressive epilepsy that was resistant to anticonvulsive treatment and unclear encephalopathy related to Hashimoto thyroiditis; all of these problems were finally ameliorated by corticoids (33). Another girl (15) with elevated thyroid antibodies and a fluctuating course of HE had no specific alterations of EEG but widespread slowing of the background activity that occurred during two such episodes. Cortical edema was indicated by cranial MRI during the first episode of encephalopathy, in the context of cerebral seizures. The patient was steroid-responsive (34). Perhaps the first pediatric case to receive immunoglobulin therapy was that of a boy who suffered from HE but responded only to intravenous immunoglobulin therapy (31).

One patient has been reported with a repeated generalized convulsive status that was resistant to various antiepileptic treatments but improved after methylprednisolone. The finding of positive thyroid antibodies called the author’s attention to the appropriate diagnosis of HE (35). Similarly, a decisive role for a positive thyroid antibody finding has been stressed after the successful treatment by intravenous corticoids of a 37-year-old male patient with a severe multifocal motoric status epilepticus, body ataxia, semirhytmic convulsions and impaired signals in the precentral cortex at MR examination (36). Possibly the first case of AIDS dementia with HE was reported in a senescent woman who was, however, resistant to steroid therapy (37). Two very rare cases of HE were also reported in which the neurological manifestations developed 10 to 20 years before the proof of autoimmune thyroiditis. In both of these cases, a dramatic responsiveness to steroids was observed, whereas a total recovery after several relapses was confirmed 3 years after the end of treatment (38).

Progressive spastic paraparesis and a hyperintense signal in the spinal cord and brain were observed in a 35-year-old woman in whom the muscle biopsy showed perivascular lymphocytes around endomysial vessels, but she had also primary hypothyroidism and a high level of thyroid antibodies, thus supporting the association of the spinal cord involvement and abnormal muscle biopsy with HE (39). An old male with seizures, progressive cognitive deterioration and in whom anti-epileptic treatment was not effective considerably improved after 6 months of treatment with prednisolone. Thus, it was recommended to use HE as a differential diagnosis in cases of epilepsy in the elderly, because antiepileptic drug treatment is only successful in 60–70% of adult-onset epilepsy (40). Although antipsychotic therapy has not been previously described in the pediatric population with HE, it appeared useful (risperidone) in a 11-year-old girl who presented with features typical for HE (positive TPOab, high TSH and low FT4) together with encephalopathy, seizures and neuropsychiatric symptoms (41). Based on a case of a 38-year-old female with rheumatoid arthritis who was treated for several years with psychotropic drugs for depressive symptoms, psychotic-like manifestations and impaired cognitive function with positive EEG findings and who finally improved considerably after corticosteroid treatment, it was recommended to suspect HE in young females with a history of autoimmune disorders and EEG abnormalities (42). An unusual case of a 37-year-old woman with persistent high fever, confusion, neck stiffness, anterograde and retrograde amnesia and disorientation with positive MR findings and a tentative diagnosis of non-herpetic acute limbic encephalitis was also reported. In addition, she had mild hypothyroidism and positive TPOab and TGab, but also antibodies against amino terminal alpha-enolase. Her condition improved after prednisolone (43). An unusual female patient presented with goiter, recurrent encephalopathy and elevated TPOab, but apparently responded to steroid therapy. Her magnetic resonance imaging was atypical for HE, and she was diagnosed with MELAS syndrome (mitochondrial encephalomyopathy, lactic acidosis, and stroke-like episodes). Although such syndrome can present with apparent steroid-responsive encephalopathy and also with elevated thyroid peroxidase antibodies, thereby mimicking HE, it was stated that even in such cases MELAS should be suspected if stroke-like episodes are present together with lactic acidosis in the cerebrospinal fluid and blood and typical features are detected by MRI (44).

A female (55 years) with type 1 diabetes presented with an acute comatose state two years after renal transplantation that appeared to be HE. Although HE has not been reported in the renal recipient populations, the authors discussed the possibility that the increased use of steroid-free immunosuppression after renal transplantation as well as the intensive lymphocyte depletion regimen possibly increased the opportunity for de novo autoimmune disease onset (45). Lithium-induced HE in a 61-year-old woman with a type II bipolar disorder has been also reported. She had positive thyroid antibodies and was effectively treated by prednisolone (46). In a cohort of 10 HE patients, the neurological targets of TPOab were explored. Their sera and anti-TPO monoclonal antibodies were able to bind cerebellar cells expressing glial fibrillary acid protein as well as normal human astrocytes from primary cultures, which suggests a role of these Abs in HE pathogenesis (47).

Two patients with Down syndrome and HE improved after treatment with corticosteroids (48). A unique case of insidious onset and slow progression of ataxia was observed in a 41-year-old woman who could no longer stand or walk without support but did not have any other neurological findings except mild EEG abnormality. She showed euthyroidism, and anti-NAE antibodies were strongly positive. After 3 weeks of steroid treatment, her ataxia markedly improved and she was able to walk unaided (49). Serum autoantibodies against the NH2-terminal of a-enolase (NAE) were analyzed in 84 cases of HE (26 men and 58 women aged 19–87 years) from several countries. Among them, 37 patients (44%) were positive. Most patients were euthyroid, and all had positive TPOab and TGab; some also had TRab. Only 20% of them had a past history of HT. The acute encephalopathy form showed various features. Abnormalities in EEG and elevated proteins/IgG in CSF were common, whereas MRI abnormalities were rare. Immunotherapy (glucocorticoids, immunosuppressants, immunoglobulin, plasma exchange) was effective (50).

A 42-year-old woman presented with acute febrile status. She had alopecia since 13 years of age (51) and Hashimoto thyroiditis since 8 years prior to the case report. Since 2 years prior to the case report, she also was progressively tired with insomnia, cramps, tremors, athralgia, back pain, and paraesthesia of the hands as well as behavioral changes and agitation that resulted in psychiatric treatment. Her mother, two sisters, and a daughter have Hashimoto’s thyroiditis, and one sister also has vitiligo. Over the next 2 months, she lost the nails of her right hand and had lesions on her back and face compatible with excoriation. Pulse therapy with methyl predinisolone (1 g i.v./day) for 3 days gave excellent results and she was maintained on oral prednisolone.

Thus far, only a few HE cases have been reported to present with pure psychiatric syndromes. Thus, one 73-year-old woman with a history of autoimmune thyroiditis had psychotic symptoms for three years that responded poorly to antipsychotic agents. After the diagnosis of HE was made, the psychotic symptoms subsided completely in a few days after high-dose intravenous steroid therapy, although the patient still presented neurologic symptoms and signs including abnormal electroencephalography. The neuropsychiatric manifestation of HE can be similar to that of typical schizophrenia (52). A young man (18 years) with stroke (!), fever, headache, and neuropsychological symptoms presented with clinical, neuroradiologic, and cerebrospinal fluid findings of cerebral small vessel vasculitis. After extensive investigation and exclusion of other causes of stroke, the diagnosis of HE was validated and corticosteroid treatment resulted in a prompt remission and a good recovery (53).

A 48-year-old woman had progressively declining cognitive function over 6 weeks without neurologic focal deficit. MRI showed cortical and subcortical white matter demyelination. Although her thyroid function tests were normal, thyroid antibodies were detected in high titers in her serum. HE was diagnosed, and over 8 weeks of corticoid treatment, her Mini-Mental State Examination Score improved to 24/30 and MRI changes showed resolution paralleling her clinical improvement (54). Long-term recovery (7 years) from repeated seizures, personality changes, and unsteadiness in gait was observed in a woman after intravenous immunoglobulin (55). A woman (38 years old) with HE showed impaired cognitive function, and the somatosensory evoked potentials and motor evoked potentials showed abnormalities of the central nervous system. Although steroid pulse therapy with resulting oral prednisolone treatment resulted in the remission of symptoms, the results of the electrophysiological tests remained unimproved (56).

In a 52-year-old woman, neuroimaging changes were described, such as bilateral multifocal abnormalities in the cortical and subcortical areas that rapidly disappeared with marked improvement of the clinical symptoms. It is therefore necessary to assess thyroid antibodies in patients with rapidly progressive cognitive dysfunction irrespective of the history of thyroid function abnormalities (57). Another woman with a history of thyroid disease presented with generalized seizures, a rapid decline in cognitive function, increasing dependency and a gradual change in personality. High thyroid autoantibody levels confirmed HE, and the symptoms improved with prednisolone. Thus, HE should be considered in cases of presenile dementia, particularly in patients with a history of thyroid disease (58).

## 7. Rare Cases of HE With Hyperthyroidism as Published Thus Far

It seems of special interest that the majority of HE cases published thus far were in the hypothyroid or euthyroid state, which appears to result from the presence of TPab or TGab or both, which contribute to the destruction of thyrocytes and lead to deficient production of thyroid hormone and thus to hypothyroidism. However, only a few HE patients presented thus far with hyperthyroidism (59). The possibility of hyperthyroidism should be considered to prevent diagnostic problems. In addition, the term “Hashimoto encephalopathy” is misleading, because it suggests thyroid hypofunction.

It is generally considered that the first patient with HE accompanied by Graves’ disease who responded to corticoid treatment was described in 2000 (5); however, a similar hyperthyroid case was actually already published one year earlier (60). Later, some further cases of HE associated with hyperthyroidism were found. Among them was a 23-year-old woman with Graves’ disease and generalized convulsions, disturbance of consciousness and hyperthyroidism (tachycardia, positive TPOab in the serum and CSF). After one month of anticonvulsant and antithyroid therapy, the patient developed a second convulsion episode as well as a cognitive deficit in her short-term memory, and there were also positive findings on EEG and SPECT. After treatment by steroids, her status improved, and the TPOab disappeared in the CSF and decreased in the serum (61). Chong (2003) in a literature review found only 4 patients with hyperthyroidism among 85 HE patients (3). At the same time, another HE patient with hyperthyroidism was presented (62). A 79-year-old female presented with unconsciousness and convulsions, myoclonic movements of face and right fingers, and tremors in the right arm and leg. She had increased TPOab, TGab, TRab, and NAE antibody. Laboratory tests revealed hyperthyroidism with increased TRab. Because after the initial treatment of hyperthyroidism she did not regain consciousness, HE was suspected, and prednisolone treatment alleviated her symptoms and normalized the EEG findings (63). Recently, a 31-year-old female with thyrotoxic HE was reported whose daughter has been followed up with the same diagnosis. Suboptimal improvement was observed with intravenous methylprednisolone, intravenous immunoglobulin and plasmapheresis. Decreased blood level of thyroid antibody resulted in an improvement of status, but she relapsed under oral immunosuppressive therapy. Full recovery was achieved only after thyroidectomy. These data may contribute to the clarification of pathogenetic role of thyroid antibodies in HE. Thyroidectomy may be considered as a treatment option, especially in thyrotoxic HE patients with uncontrolled relapses. In addition, this patient was possibly the first reported HE case with a family history (64). Genetic background can contribute to the etiopathogenesis of HE, as also known for other cases of autoimmune disorders. A similar case was also mentioned above (5), whereas another case developed after thyroid radiotherapy with iodine-131 for Graves’ disease (65).

A 23-year-old Malay woman who initially presented with status epilepticus was empirically cured with i.v. antibiotics for meningitis and levetiracetam for the seizures. Later, she presented with a 2-month history of altered behavior and seizures. She had received the diagnosis of Graves’ disease previously but was not compliant with treatment. A diagnosis of thyrotoxic psychosis was subsequently made, and she was given risperidone. Although she was discharged a month later, she was still unable to work because of deterioration in her mental function. She continued to have weekly seizures. She also had a diffuse goiter, exophthalmus, and was clinically hyperthyroid. After several examinations, she was admitted to another center where extremely high TGab and TPOab levels were found. Thus, the diagnosis of HE was made, and within 48 h of intravenous glucocorticoid administration, there was a dramatic improvement that included the mental status (66).

We also observed a 27-year-old woman who apparently also belonged to that sprinkling of HE cases with hyperthyroidism (67). Approximately four months before the acute onset of neural symptoms, the endocrinologist found low TSH, high FT4 and high thyroperoxidase antibodies (TPOab) and prescribed treatment by carbimazol. However, after a few weeks, she deliberately withdrew from this drug, and neural symptoms started after 5 days of febrile status with headache and arthralgia treated by an antibiotic. At that time, her husband noticed irregular breathing and responsiveness only to painful stimuli. The emergency physician found roving eye movements, intermittent seizures and altered consciousness. She was admitted to the hospital and subjected to artificial pulmonary ventilation. She had negative brain CT, increased C-reactive protein, decreased TSH level (0.02 mU/l), and increased FT4 level (53.6 pmol/l), which clearly showed thyrotoxicosis. The level of all thyroid antibodies (TPOab, TGab and TRab) was very high. CSF showed increased IgG, IgM, albumin, and total protein. Starting on the first day after the admission, she was treated by carbimazole (30 mg/d) and hydrocortisone (200 mg/d). After 6 days, her status was stabilized and hydrocortisone was replaced by prednisone (15 mg/d). However, three days later, her status was aggravated, and the consulting psychiatrist concluded that this was possibly the resulted of side effects of the corticoid treatment. However, after the withdrawal of the corticoids, her status rapidly worsened. Hydrocortisone was started again (300 mg/d), which resulted in a nearly complete restitution of psychological function within 3 days. Nevertheless, after the next withdrawal of corticoids, the progressive aggravation of psychological functions and epileptic-like seizures appeared again. We finally concluded that her symptoms, including thyrotoxicosis with low TSH, high FT4, and thyroid antibodies, as well as repeated worsening of the condition after withdrawal from corticoids, were compatible with the diagnosis of HE. Over the next three months, we gradually discontinued the corticosteroid medication and, because of the development of hypothyreosis, we discontinued carbimazol and started with levothyroxin treatment. A thin-needle biopsy showed signs of chronic exacerbated Hashimoto thyroiditis. SPECT examination showed normal accumulation in both hemispheres. Her long-term status now appears fully stabilized.

Although the majority of the described cases showed neural symptoms for months before the acute onset, in this case we admitted a dramatic acute onset. Thus, neurologic complications can begin either abruptly, in the form of seizures or agitation, with or without other neurologic complaints, or they can develop gradually, in a relapsing-remitting manner, including, among others, cognitive deterioration and psychiatric illness.

## 8. Conclusions

HE is a rare but very serious illness. The incidence is probably underestimated because of the low overall awareness about the disease. HE may be found in cases of unexplained encephalopathy, particularly together with the presence of high thyroid antibody levels, especially against thyreoperoxidase (TPOab). Because of the autoimmune origin of the disease, corticosteroid treatment usually provides a dramatic recovery.
